# Smiles or struggles? How trust (in)congruence influences subordinates’ ambivalent relational identification and upward ingratiation?

**DOI:** 10.3389/fpsyg.2025.1610495

**Published:** 2025-11-05

**Authors:** Xiaodong Ma, Minmin Zhang

**Affiliations:** ^1^Business School, Central University of Finance and Economics, Beijing, China; ^2^The Army Infantry College of PLA, Shijiazhuang, Hebei, China

**Keywords:** expected leader trust, perceived leader trust, upward ingratiation, ambivalent relational identification, rising ridge congruence asymmetry

## Abstract

Recent research on trust in organizational behavior has largely centered on perceived leader trust (PLT), shedding light on how being trusted influences employee behavior. However, this focus has often neglected expected leader trust (ELT)—employees’ internal expectations of being trusted—thus limiting insight into behavioral differences and the psychological mechanisms driven by trust discrepancies. To address this gap, the present study incorporates both ELT and PLT to provide a more holistic understanding of subordinates’ psychological dynamics and behavioral responses in trust relationships. Grounded in relational identity theory, we investigate how distinct trust configurations affect upward ingratiation (UI) and examine the mediating role of ambivalent relational identity (ARI). Employing a mediated Rising Ridge Congruence Asymmetry approach, we analyzed three-wave dyadic data from 330 supervisor–subordinate pairs. The findings reveal that: (1) UI is significantly lower when ELT and PLT are aligned; (2) When trust discrepancy is held constant, higher overall trust levels—particularly high ELT—are associated with increased UI; (3) Given the same average trust level and magnitude of discrepancy, UI is more pronounced when ELT exceeds PLT than when PLT exceeds ELT; (4) Across all trust configurations, ARI significantly mediates the relationship between ELT–PLT configurations and UI, indicating that identity conflict stemming from trust misalignment is a key psychological mechanism behind strategic ingratiation. This study extends the theoretical scope of trust research, offers deeper insight into its dynamic nature, and provides new empirical support for applying relational identity theory in trust-related contexts.

## 1 Introduction

Trust has long been recognized as a fundamental mechanism for enhancing organizational effectiveness in the field of organizational behavior (Song et al., 2025). However, with the widespread adoption of artificial intelligence, the normalization of remote collaboration, and the increasing pressure for sustainable development, organizations are undergoing profound transformations in the way they operate (Vuori et al., 2025). Leaders are now required not only to unleash employees’ potential and foster team collaboration in highly uncertain and competitive environments (Liu et al., 2025), but also to rebuild trust grounded in transparency and accountability amid technological disruption and shifting value orientations (Demartini et al., 2025).

Meanwhile, an emerging stream of research highlights that subordinates are not merely passive recipients of trust but can actively engage in behaviors to earn their leaders’ trust, thereby shaping their roles and developmental trajectories within organizations (Morrison, 2011). However, extant studies on organizational trust have predominantly focused on top-down trust-building processes—such as empowerment, transparent communication, and risk-taking (Rosenbruch et al., 2023)—while overlooking the inherently interactive and reciprocal nature of trust between supervisors and subordinates. Moreover, although existing studies have paid attention to the role of felt trust in interpersonal trust formation (de Jong et al., 2025), the role of expected trust has been largely overlooked (Baer et al., 2021; Mignonac et al., 2025).

With the increasing application of social exchange theory in trust research, scholars have gradually shifted from viewing trust as a unilateral managerial decision to conceptualizing it as a dynamic, co-constructed process rooted in role expectations and interpersonal interactions (Brower et al., 2009). Subordinates may proactively participate in trust-building by demonstrating competence, assuming responsibility, and engaging in relationship maintenance behaviors (Colquitt et al., 2007). This perspective challenges the traditional assumption that trust flows unidirectionally from leader to subordinate, driving a theoretical transformation of trust research from static to dynamic, and from one-way to reciprocal processes (Detert and Burris, 2007).

Building on this shift, a growing body of literature has begun to examine how subordinates engage in strategic behaviors to influence their supervisors (Wang and Luan, 2024; Xiao et al., 2025). Among these, upward ingratiation (UI)—a common impression management tactic—refers to subordinates’ efforts to shape favorable images in the eyes of their leaders by expressing loyalty, offering flattery, and aligning with their preferences (Kipnis et al., 1980; Bolino et al., 2016; Long, 2021). Studies have shown that UI can enhance an employee’s visibility and performance evaluations (De Clercq et al., 2021), and is positively associated with career outcomes such as promotion and compensation (Gross et al., 2021). Although the critical role of subordinates’ UI tactics in organizational dynamics has been widely acknowledged, existing research still lacks sufficient exploration into the mechanisms that trigger such behaviors—particularly how subordinates adjust their strategies within specific relational contexts (Kacmar et al., 2004). A meta-analysis conducted by Barbuto and Moss (2006) highlighted a structural bias in the literature on upward influence, noting a predominant focus on individual-level antecedents, such as locus of control, political skill, and identity orientation, while giving insufficient attention to interpersonal dynamics embedded in leader–subordinate interactions. This suggests that UI, as a specific form of upward influence, has been primarily understood through a dispositional lens, overlooking the relational context in which it unfolds (Barbuto and Moss, 2006).

More recently, a small but growing body of research has begun to examine how leaders’ perceptions influence subordinates’ strategic behaviors. For example, misalignments in leader expectations—such as those concerning empowerment—have been found to significantly shape subordinates’ upward influence tactics, indicating that such behaviors are not solely driven by stable personality traits but also reflect adaptive responses to leaders’ actions and expectations (Wong, 2019). These findings suggest that UI is deeply embedded within the relational dynamics of leader–follower interactions and is contingent upon the degree of cognitive alignment within those relationships.

However, much of the existing work remains rooted in a leader-centric perspective, providing limited understanding of how subordinates themselves perceive trust and regulate their behavior accordingly. To address this limitation, recent research has adopted a cognitive matching approach, exploring how the consistency between subordinates’ expected and perceived levels of trust influences their fairness perceptions and subsequent work outcomes (Baer et al., 2021). This research shows that alignment between trust expectations and perceptions fosters a greater sense of fairness and positive organizational outcomes, whereas misalignment may result in psychological strain and adverse behavioral responses. These findings underscore the importance of cognitive congruence as a key mechanism linking relational perceptions to strategic behavior in organizations.

To set the stage for the proposed framework, it is necessary to recognize two fundamental gaps in existing research on UI. First, prior studies have largely neglected the relational foundation of leader–subordinate interactions. As a strategic form of interpersonal influence, UI is deeply embedded in the quality and dynamics of leader–subordinate relationships (Kim et al., 2022). When detached from this relational context and explained merely by individual traits or situational cues (Kacmar et al., 2004), UI becomes conceptually flattened, obscuring the deeper logic of why and how employees engage in such behavior.

Second, existing research tends to portray employees as passive reactors rather than strategic agents. Although trust alignment has been shown to affect employee responses, most studies rely on fairness-based models that depict subordinates as recipients of organizational treatment (Baer et al., 2021). This perspective overlooks employees’ proactive regulation of their own behavior in response to perceived trust discrepancies (Wong, 2019). In reality, employees often evaluate and strategically adjust their actions to maintain or restore relational trust. Accordingly, a new framework that integrates both relational embeddedness and agentic intentionality is needed to capture the generative mechanisms of UI and explain its diverse manifestations (Hou et al., 2024).

To address these theoretical gaps, this study adopts Relational Identification Theory (RIT) (Sluss and Ashforth, 2007) as its foundational framework and introduces trust consistency as a critical cognitive congruence variable. This theory posits that subordinates construct their organizational identity not only through self-definition but also through their identification with others—particularly their direct supervisors. Unlike fairness-based theories, which portray subordinates as passive evaluators of environmental cues, relational identification theory emphasizes that subordinates are active agents who strategically build and regulate interpersonal relationships within organizations (Lee et al., 2015).

Within this framework, trust consistency—the employee’s subjective judgment regarding the alignment between expected leader trust (ELT; Lau et al., 2007, 2014) and perceived leader trust (PLT; Baer et al., 2021; Mignonac et al., 2025)—is conceptualized as a core cognitive mechanism that activates relational identification. This study argues that trust consistency not only shapes subordinates’ evaluations of the leader–subordinate relationship but also configures the pattern of their relational identification, which subsequently drives behavioral responses. Specifically, subordinates may engage in ingratiation behavior via two theoretically distinct pathways.

On the one hand, a motivational compensation pathway may be activated when subordinates expect to be trusted but perceive a lower level of actual trust (Hao et al., 2021). This discrepancy triggers cognitive dissonance, heightens role ambiguity, and increases psychological uncertainty, thereby weakening subordinates’ sense of relational security and diminishing their identification with their supervisor. To bridge this trust gap and regain recognition, subordinates may proactively engage in ingratiation behavior as a compensatory strategy to reinforce relational bonds. Conversely, when expected and perceived trust are well aligned, subordinates experience a clearer and more stable relational identity, enhanced psychological safety, and consequently, a diminished need to engage in such strategic behavioral regulation. From this perspective, ingratiation tendencies are expected to decrease as trust consistency increases.

On the other hand, a relational identity tension pathway may emerge when trust inconsistency not only reduces relational identification but also gives rise to a conflicted identification state—what this study refers to as ambivalent relational identification (Ashforth et al., 2014). According to RIT, relational identification is shaped not merely by its strength but also by the perceived clarity, coherence, and stability of the relationship. When subordinates perceive a substantial gap between expected and actual trust, they are likely to experience conflicting emotions, such as a simultaneous desire for closeness and a sense of psychological distance. This emotional ambivalence creates a state of internal identity conflict, wherein subordinates feel “neither able to approach nor able to withdraw” from the leader. In response, they may resort to ingratiation as a strategy to test, stabilize, or repair the relationship, thereby restoring a sense of psychological coherence and relational clarity.

In this context, ingratiation operates not solely as a means of impression management, but also as a form of identity regulation—a deliberate response to relational ambiguity and emotional dissonance. Unlike traditional relational identification processes, ambivalent relational identification offers a more nuanced explanation for the psychological strain induced by trust inconsistency and its downstream behavioral manifestations (Schuh et al., 2016; Rothman et al., 2017; Ciampa et al., 2021).

Accordingly, this study incorporates ambivalent relational identification (ARI) as a mediating mechanism in the theoretical model to illuminate the identity-based processes through which trust consistency influences ingratiation behavior (Sluss and Ashforth, 2007). This perspective complements the compensatory pathway by emphasizing the roles of identity pressure and emotional contradiction in shaping strategic behavior within organizational interactions.

**Figure 1:**
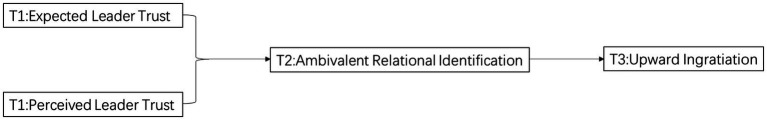
Conceptual model. T1 and T2 were rated by subordinates, while T3 was rated by supervisors.

In summary, by focusing on how subordinates interpret and respond to discrepancies between expected and perceived trust, this study aims to extend the literature on organizational trust and impression management from a bottom-up perspective. In contrast to the dominant top-down paradigm—which emphasizes leader-initiated trust and employee passivity—this research challenges the conventional assumption that subordinates are merely passive recipients in the trust-building process. It underscores the active role of subordinates as interactive participants in constructing trust relationships. This theoretical shift not only provides a new explanatory lens for upward influence behaviors but also sheds light on the micro-foundations and dynamic evolution of organizational trust. The conceptual model is shown in Figure 1, and it will be described in detail in the following sections.

## 2 Hypotheses and literature review

### 2.1 ELT, PLT and UI

According to RIT (Sluss and Ashforth, 2007), subordinates’ relational self-identification within organizations is shaped by their interactions with significant others, such as direct supervisors. This identification is particularly influenced by whether subordinates perceive acceptance and recognition from these figures. When subordinates perceive the level of trust received from their leaders (i.e., perceived trust) as congruent with their own expectations of trust (i.e., expected trust), relational identification tends to stabilize. In such a “trust congruence” scenario, subordinates perceive alignment between their expectations and the leader’s attitude toward them, which fosters a sense of psychological safety. As a result, they are less motivated to engage in compensatory behaviors aimed at repairing the relationship or reinforcing their identification. Instead of resorting to strategic impression management tactics—such as UI—subordinates interact with their supervisors in a more authentic and natural manner.

Lau et al. (2014) also argue that when subordinates feel appropriately respected and trusted within organizational relationships, they are more likely to engage in honest and non-strategic behavior (Lau et al., 2014). Trust congruence helps mitigate role conflict and uncertainty, reducing subordinates’ motivation to seek approval or avoid risk through ingratiatory behavior. Thus, trust congruence provides psychological support for relational identification and inhibits UI tendencies.

In contrast, trust incongruence may disrupt subordinates’ relational identification, thereby increasing the likelihood of UI. First, when ELT is higher than PLT, subordinates may feel undervalued, which triggers compensatory identification motives. To restore their self-worth, they may engage in flattery or other pleasing behaviors. Bolino et al. (2008) also suggest that when subordinates sense that trust resources are limited, they are more inclined to adopt impression management strategies to improve their evaluations by supervisors (Bolino et al., 2008).

Second, in a “low expected—high perceived” scenario, subordinates receive more trust than they anticipated, which may lead to feelings of responsibility and role overload. To avoid disappointing their leaders, subordinates may adopt strategic behaviors—such as ingratiation—to maintain positive evaluations. This “downward stabilization” motivation reflects subordinates’ psychological need to sustain an already favorable trust dynamic (Grant and Wrzesniewski, 2010).

In sum, congruence between ELT and PLE fosters stable relational identification and reduces UI. In contrast, any form of incongruence—whether ELT exceeds or falls short of PLE—may trigger UI due to identification anxiety or trust-related stress. Based on this reasoning, we propose the following hypothesis:

*H*1: Employees are less likely to engage in UI when ELT aligns with PLT.

Even under trust congruence, the overall level of trust (i.e., the average of ELT and PLT) may independently influence employee behavior. RIT posits that the higher the quality of one’s relationship with significant others, the more likely the individual is to activate a relational self-concept and engage in proactive behaviors to maintain that relationship (Sluss and Ashforth, 2007). In high-trust situations, subordinates may feel a stronger sense of responsibility tied to their role within the relationship, which motivates them to engage in behaviors—such as UI—to uphold the leader’s positive impression.

Conversely, in low-trust congruence scenarios (i.e., low perceived–low expected), although no trust gap exists, the overall level of trust is relatively low. This results in a weaker sense of relational value, limiting the employee’s motivation to engage in extra efforts to preserve the relationship. Prior research also suggests that when positive identification mechanisms are lacking between subordinates and leaders, behaviors tend to become more defensive and disengaged (Epitropaki and Martin, 2005).

Furthermore, within trust congruence scenarios, high ELT may exert a particularly strong influence. When subordinates perceive that their supervisors hold high expectations for trust, they often experience heightened role responsibility and concern about failing to meet such expectations. As a result, they may engage in “preventive ingratiation” as a strategy to maintain a positive relational evaluation. This “role-enhancement pathway” illustrates how subordinates adjust their behavior in response to trust expectations in order to solidify their positive identification role.

Therefore, even when ELT and PLT levels are aligned, the overall trust level can still shape impression management motivations and behavioral strategies. Accordingly, we propose the following hypothesis:

*H*2: Controlling for trust congruence, higher overall trust levels are associated with greater upward UI, particularly when ELT is high.

When holding the average trust level and degree of trust discrepancy constant, the direction of trust incongruence may differentially influence employee behavior. RIT suggests that when subordinates perceive themselves as failing to meet their leaders’ relational expectations (i.e., ELT is higher than PLT), they are likely to activate self-regulatory mechanisms and adopt strategic behaviors, such as UI, to address the identification gap.

In “high ELT–low PLT” scenarios, subordinates recognize that their leaders expect a high degree of trust, yet they do not perceive corresponding trust support. As a result, they may view themselves as falling short of the expected relational role, which elicits identity threat. In such cases, UI becomes a key strategy to restore the leader’s positive evaluation and close the subjective-objective trust gap.

To be sure, “high PLT–low ELT” scenarios may also generate responsibility-related pressure, prompting subordinates to engage in UI for the sake of maintaining the status quo. However, compared to the “high ELT–low PLT” context, the latter imposes a greater sense of identity threat and pressure, as subordinates confront higher unmet expectations in the absence of sufficient trust. Consequently, UI is more likely to be activated as a compensatory and restorative strategy.

This mechanism is also supported by psychological contract violation theory. Research has shown that when subordinates fail to meet others’ high expectations, the resulting stress and sense of responsibility tend to be more intense than in “low ELT” scenarios (Coyle-Shapiro, 2002). Therefore, the “high ELT–low PLT” condition is more likely to trigger identification repair motivations, which in turn lead to UI.

Based on this reasoning, we propose the following hypothesis:

*H*3: Given equal levels of average trust and trust discrepancy, ELT higher than PLT is more likely to elicit UI than the reverse.

### 2.2 ELT, PLT, UI and ARI

In the preceding discussion, we highlighted the importance of congruence between ELT and Perceived PLT in shaping subordinates’ strategic behaviors. However, trust levels do not operate in isolation; rather, the psychological mechanisms triggered by trust congruence warrant further theoretical exploration. To gain a more comprehensive understanding of how this congruence affects UI, we introduce the concept of ARI as a key explanatory mechanism.

ARI refers to the psychological state in which individuals experience simultaneous attraction and aversion toward defining themselves through a particular relationship (Ashforth et al., 2014). In the leader-employee context, this manifest when subordinates both desire and resist deriving self-definition from their relationship with their leader, due to relational asymmetry, ambiguity, or perceived risks (Schuh et al., 2016). Unlike clear-cut relational identification or disidentification, ARI is characterized by internal tension and conflicting motivations. According to RIT, subordinates tend to construct their work identity through the relational roles they occupy, and the clarity and consistency of these relationships are central to forming a stable self-concept.

When subordinates perceive alignment between ELT and PLT, they are more likely to experience a stable and coherent relational identity, which reduces cognitive conflict and emotional ambivalence. Under such conditions, individuals are less compelled to engage in strategic behaviors to manage impressions or relational uncertainty. Conversely, when there is incongruence between ELT and PLT, relational ambiguity arises. In the “high ELT–low PLT” condition, subordinates may perceive themselves as undervalued or insufficiently trusted, prompting compensatory behaviors aimed at meeting expectations. In contrast, under the “low ELT–high PLT” condition, subordinates may feel over-trusted relative to leader expectations, leading to concerns about sustaining their current status. In both cases, relational inconsistency may elicit anxiety, thereby increasing the likelihood of strategic behaviors such as flattery or ingratiation.

In short, trust congruence is not only a matter of cognitive evaluation but also a relational signal that shapes identity construction. When ELT and PLT are misaligned, subordinates are more likely to experience ambivalent relational identification, which in turn motivates UI as a coping strategy to restore balance and reduce psychological tension (Kwang and Swann, 2010).

Based on this reasoning, we propose the following hypothesis:

*H*4: The congruence between ELT and PLT influences UI via ARI.

## 3 Methods

### 3.1 Sample collection

This study recruited subordinates and their immediate supervisors from the service, technology and finance, and manufacturing industries, with data collected in three waves from 73 teams. To minimize common method bias, we employed a three-wave, multi-source design: T1—expected and perceived leader trust (ELT/PLT); T2—employee upward ingratiation and role perception (ARI/role perception); T3—leader evaluation of upward ingratiation (UI), with approximately 14-day intervals between waves. At the outset, the research team informed HR managers of the study’s objectives and academic significance and assured strict confidentiality. With organizational consent, HR managers provided subordinate lists and facilitated supervisor contact, ensuring smooth questionnaire distribution and collection.

In Phase 1, 80 teams completed surveys measuring ELT and PLT (381 valid responses). In Phase 2, subordinates completed ARI surveys, yielding 359 valid responses. Finally, in Phase 3, supervisors assessed subordinates’ UI, producing 330 valid responses. Among subordinates, 43.6% were female, and 29.7% were aged 26 or above. Approximately 40% held a bachelor’s degree or higher. Regarding organizational tenure, 37% had been with the organization for more than 3 years, while 63% had 3 years or less. Among supervisors, 47.9% were female, and 45.2% were aged 35 or below. Half of the supervisors (50%) held a bachelor’s or master’s degree. In terms of tenure, 17.9% had worked for 3 years or less, 36.4% for 4 to 6 years, and 45.8% for more than 7 years. Factorial ANOVA indicated no significant team effects; nevertheless, multilevel modeling was employed to control for leader demographics and other potential confounds, ensuring robust estimation of cross-level relationships.

### 3.2 Variable measurement

To ensure the reliability and validity of the measurements, all scales used in this study were adapted from established sources published in top-tier academic journals. All variables were measured using a five-point Likert scale ranging from 1 = “strongly disagree” to 5 = “strongly agree.”

ELT and PLT were assessed following best practices for evaluating expectation–perception (E-P) congruence. Specifically, we adopted an atomistic approach by using parallel items to measure personal expectations (i.e., ELT) and environmental supplies (i.e., PLT). In line with Baer et al. (2021), both ELT and PLT were measured using the behavioral trust scale developed by Gillespie (2011). Each scale consisted of five items. A sample item for ELT was: “I hope my supervisor is willing to share his/her emotions and feelings with me.” A sample item for PLT was: “My supervisor is willing to share his/her emotions and feelings with me.” The Cronbach’s *α* coefficients were 0.910 and 0.900 for ELT and PLT, respectively.

ARI was measured by adapting the Ambivalent Organizational Identification Scale (Kreiner and Ashforth, 2004), with the referent modified to reflect supervisor-subordinate relational identification. The final ARI scale included six items. A sample item was: “On the one hand, I recognize my supervisor; on the other hand, I feel dissatisfied with him/her.” The Cronbach’s α coefficient for this scale was 0.907.

UI was assessed using a four-item scale originally developed by Bolino and Turnley (1999). The items were slightly revised to be completed by supervisors in order to evaluate subordinates’ ingratiatory behaviors. A sample item was: “This employee often shows concern for my personal life or emotions, behaving in a friendly and warm manner.” The Cronbach’s α coefficient for this scale was 0.902.

### 3.3 Analytical strategy

Prior research on congruence hypotheses has predominantly employed quadratic response surface analysis (RSA; Edwards, 1994). However, such models fall short in detecting directional asymmetries in incongruence effects. Although congruence (e.g., ELT = PLT) may yield either optimal or suboptimal outcomes, the two incongruent conditions (e.g., ELT > PLT vs. ELT < PLT) can have distinct effects on UI in both direction and intensity—patterns that quadratic models are not designed to capture.

To address this limitation and in line with the methodological recommendations proposed by Humberg et al. (2022), the present study adopts a third-order polynomial regression combined with the Rising Ridge Congruence Asymmetry (RRCA) approach to more accurately capture the nuanced effects of ELT and PLT on UI.

Although the RRCA model offers a more robust framework for assessing both congruence and asymmetry effects, methods for testing mediation within this model remain underdeveloped. Previous studies have commonly applied the block variable approach to examine the mediating role of congruence, yet this method faces notable limitations when dealing with multiple forms of congruence or parallel mediation pathways.

To address these limitations, this study integrates cross-level polynomial regression (Chaudhry et al., 2021) with the RRCA framework (Humberg et al., 2022) to construct a mediated RRCA model (Humberg et al., 2022) for testing the proposed theoretical mechanism. The PLT and ELT variables were jointly mean-centered, and quadratic and cubic terms were subsequently generated based on the centered data to improve the precision of model estimation and enhance the interpretability of the results.

### 3.4 Our study adopted the following statistical analysis strategy

First, confirmatory factor analysis (CFA) was conducted using Mplus 8.3 to examine the discriminant validity of the main constructs and to assess the potential threat of common method bias.

Second, descriptive statistics, correlation analyses, and reliability tests were performed using SPSS 23.0 to explore the basic relationships among variables and to assess the internal consistency of the measurement instruments.

Third, based on the theoretical model and prior derivations, a mediated response surface model (mediated RRCA model) was constructed and tested using Mplus 8.3 to investigate the complex interactive relationships among variables.

Finally, given that the mediation effects were derived from the product of multiple regression coefficients, the Monte Carlo resampling method was employed with 20,000 iterations to generate 95% confidence intervals (CIs) for testing the significance of the indirect effects (Preacher and Selig, 2012).

## 4 Results

### 4.1 Confirmatory factor analysis

Table 1 presents the results of the confirmatory factor analysis (CFA) conducted in this study. As shown in Table 1, the hypothesized four-factor model demonstrated the best fit compared to alternative competing models (χ^2^ = 218.996, df = 164, RMSEA = 0.032, CFI = 0.987, TLI = 0.985, SRMR = 0.032). These results suggest that the core variables in this study are clearly defined, distinctly different from one another, and exhibit a high degree of independence, indicating good discriminant validity.

**Table 1:** Results of CFA.

Model	χ^2^	df	RMSEA	CFI	TLI	SRMR
Four-Factor Model	218.996	164	0.032	0.987	0.985	0.032
Three-Factor Model	1077.086	167	0.129	0.779	0.749	0.155
Two-Factor Model	1849.95	169	0.174	0.592	0.541	0.201
single-Factor Model	2890.104	170	0.22	0.34	0.262	0.242
CMV Model	252.263	163	0.041	0.978	0.975	0.052

### 4.2 Common method bias

Despite the use of a multi-stage, multi-source approach for data collection in this study, there may still be some potential for common method bias. To address this concern, the study adopted multiple procedural and statistical techniques to detect potential common method bias (Podsakoff et al., 2024). First, Harman’s single-factor test revealed that factors with eigenvalues greater than 1 accounted for 72.558% of the total variance, with the first factor explaining 30.201% of the variance. No significant factor was found to affect all items. Additionally, this study included an unmeasured latent method construct (ULMC) to further test for common method bias. The results showed that the model controlling for the common method factor did not significantly improve the model fit (χ^2^ = 252.263, df = 163, RMSEA = 0.041, CFI = 0.978, TLI = 0.975, SRMR = 0.052), as shown in Table 1. Collectively, these analyses suggest that common method bias in this study has been effectively controlled to a reasonable extent.

### 4.3 Correlation analysis

The descriptive statistics and correlation analysis are presented in Table 2.

**Table 2:** Descriptive statistics and correlation analysis results.

Variable	M	S. D.	1	2	3	4	5	6	7	8
Subordinate level										
1.gender	1.564	0.497	-							
2.age	1.297	0.458	0.010	-						
3.education	1.400	0.491	0.032	0.566**	-					
4.tenure	1.909	0.801	0.022	−0.001	−0.039	-				
5. ELT	3.270	1.016	0.032	0.010	0.056	0.023	(0.910)			
6. FLT	3.381	0.984	0.011	−0.047	−0.112*	0.093	0.386**	(0.900)		
7. ARI	3.317	0.933	0.030	−0.012	0.005	0.009	0.116*	−0.343**	(0.907)	
8. UI	3.302	1.127	−0.024	−0.058	0.029	−0.046	0.552**	−0.037	0.361**	(0.902)
Supervisor level										
1.gender	1.521	0.503	-							
2.age	2.616	1.150	−0.226	-						
3.education	1.507	0.503	0.041	−0.067	-					
4.tenure	2.274	0.750	−0.052	0.011	0.105	-				

Subordinates (*n* = 330), Supervisors (*n* = 73). ***p < 0.001; **p < 0.01; *p < 0.05; The values in parentheses represent the internal consistency reliability coefficients of each scale.

### 4.4 Hypothesis testing

As shown in Table 3, we first estimated three unrestricted cubic response-surface models—one with UI as the dependent variable, one with ARI as the dependent variable, and one with UI as the dependent variable including ARI as a mediator. We then tested whether these models could be reduced to the rising-ridge congruence–asymmetry (RRCA) by imposing six linear shape constraints. The Wald statistics were χ^2^(6) = 5.902, 5.748, and 8.209, respectively (all ns), indicating that the constraints are statistically compatible with the data—that is, constraining the unrestricted cubic surface to the RRCA shape does not significantly worsen model fit. On grounds of parsimony and interpretability, we therefore present the constrained RRCA models in the main text. Model-fit comparisons (AIC and BIC) are virtually unchanged, underscoring that the constrained specification achieves comparable fit while yielding directly interpretable parameters for congruence (b3), linear level (u1), and directional asymmetry (b6).

**Table 3:** Cubic RSA results.

Estimate	UI		ARI		UI	
	RRCA	Full model	RRCA	Full model	RRCA	Full model
b1	0.214***	0.395**	−0.077**	−0.226	0.235***	0.455***
b2	0.214***	0.173	−0.077**	−0.106	0.235***	0.196
b3	0.107*	0.044	0.081**	0.144**	0.092*	0.012
b4	−0.214*	−0.210**	−0.163**	−0.178**	−0.185*	−0.171*
b5	0.107*	0.069	0.081**	0.029	0.092*	0.065
b6	0.187***	0.086	0.124***	0.215***	0.164***	0.035
b7	−0.562***	−0.514***	−0.371***	−0.377***	−0.491***	−0.425***
b8	0.562***	0.565* **	0.371***	0.359***	0.491***	0.492***
b9	−0.187***	−0.182**	−0.124***	−0.120*	−0.164***	−0.159**
u1	0.428***	0.568***	−0.154**	−0.332*	0.470***	0.651***
ARI					0.214**	0.235***
E_2_	PLT = ELT + 0.381		PLT = ELT + 0.439		PLT = ELT + 0.376	
Wald Test	5.902(6)		5.748(6)		8.209(6)	
Within-R^2^	0.491***	0.500***	0.256 ***	0.271***	0.491***	0.505***
Between-R^2^	0.763	0.715	0.781	0.467	0.214	0.174
AIC	820.296	826.446	819.346	825.65	1720.95	1724.631
BIC	873.483	902.427	872.533	901.632	1823.525	1850.001

***p < 0.001; **p < 0.01; *p < 0.05. The data analysis in this study was conducted while controlling for subordinates’ and supervisors’ demographic variables. To simplify the table, the results for these control variables are not reported.

**Figure 2:**
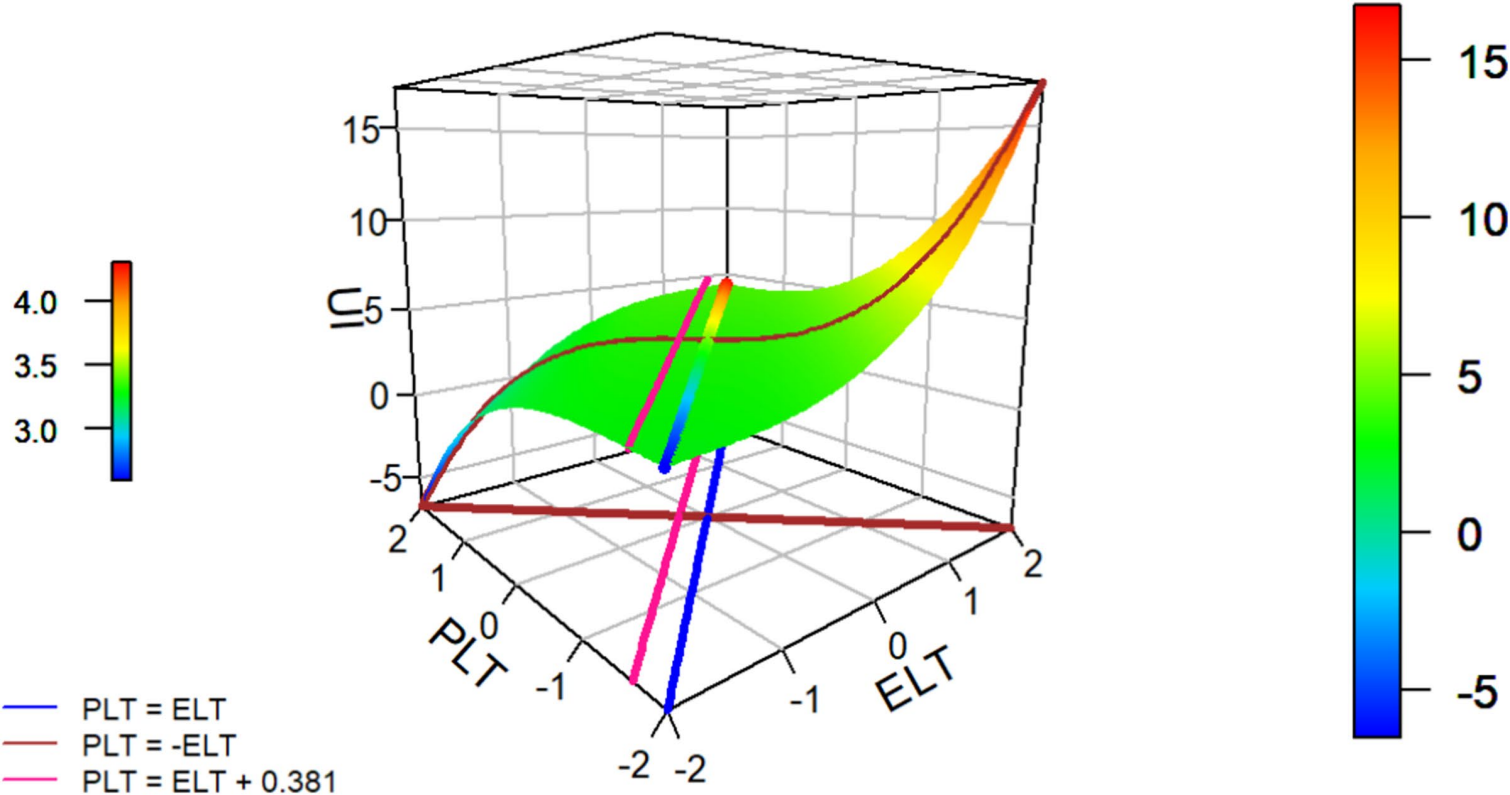
Graph of the estimated RRCA model for the UI. Blue line (LOC, PLT = ELT): UI increases as PLT and ELT rise simultaneously, reaching its peak in the high-level congruent region. Brown line (LOIC, PLT = −ELT): UI increases when ELT > PLT but decreases when PLT > ELT, indicating that the effect of trust incongruence is directionally asymmetric. Pink line (E_2_, PLT = ELT + 0.381): the second extremum line, parallel to the LOC; as one deviates from congruence toward PLT > ELT, predicted UI rises up to this line, beyond which further departures in that direction do not yield higher UI within the observed domain (treated here as a descriptive boundary).

We first examine the effects of ELT and PLT on UI. As shown in Table 3 and Figure 2, the estimated second extremum line (E2: PLT = ELT + 0.381) delineates the empirically relevant predictor region; we therefore restrict interpretation to this region. Consistent with H1, the congruence parameter is positive and significant (b3 = 0.107, *p* < 0.001). In a local neighborhood around the line of congruence (LOC; ELT = PLT), orthogonal departures from the LOC increase UI, indicating that the LOC functions as a local trough. Put differently, smaller ELT–PLT discrepancies are associated with lower UI, supporting H1.

Linear level effect (H2). Controlling for the ELT–PLT discrepancy (|ELT − PLT|), the overall trust level is positively related to UI (u1 = b1 + b2 = 0.428, *p* < 0.001). Along the LOC (ELT = PLT), moving from low–low to high–high yields a monotonic increase in UI, supporting H2.

Directional asymmetry (H3). Holding constant the mean trust level and the absolute discrepancy |ELT − PLT|, subordinates exhibit higher UI when ELT > PLT than when PLT > ELT (b6 = 0.187, *p* < 0.001). In RSA terms, the positive asymmetry parameter (b6 > 0) indicates that, starting from the LOC, the surface rises more steeply toward the ELT > PLT region than toward the PLT > ELT region, thereby supporting H3.

**Figure 3:**
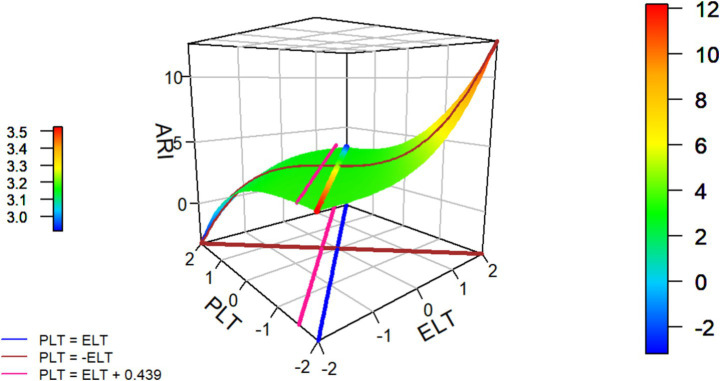
Graph of the estimated RRCA model for the ARI. Blue line (LOC, PLT = ELT): ARI decreases as PLT and ELT increase simultaneously, reaching its lowest point in the high-level congruent region. Brown line (LOIC, PLT = −ELT): ARI increases when ELT > PLT but decreases when PLT > ELT, indicating that trust incongruence has a directionally asymmetric effect on ARI. Pink line (E_2_, PLT = ELT + 0.439): the second extremum line runs parallel to the line of congruence (LOC). As values deviate from congruence toward the region where PLT exceeds ELT, the predicted ARI increases up to this line. Beyond this point, further departures in the same direction no longer yield higher ARI within the observed range, indicating that E2 serves as a descriptive boundary of the surface.

To test the mediating effect of ARI, we first examined the effects of ELT and PLT on ARI. As shown in Table 3 and Figure 2, ELT and PLT had a significant congruence effect on ARI (b3 = 0.081, *p* < 0.01), a significant linear level effect (u1 = 0.154, *p* < 0.01), and a significant asymmetry effect (b6 = 0.124, *p* < 0.001). The corresponding response surface plot is presented in Figure 3. Next, after controlling for the third-order response surface effects, ARI was found to have a significant positive impact on UI (b = 0.214, *p* < 0.01), providing preliminary evidence for the mediating role of ARI.

Additionally, we used Monte Carlo simulation (20,000 resamples) to assess the indirect effects. As shown in Table 4, the congruence (overall level) pathway yielded a significantly negative indirect effect (b = −0.033, 95% CI [−0.062, −0.004]), indicating that simultaneous increases in the overall level suppress the outcome via the mediator. By contrast, along the incongruence pathway, greater mismatch magnitude (|ELT − PLT|) produced a significantly positive indirect effect (absolute indirect effect: b = 0.017, 95% CI [0.001, 0.034]). We also observe pronounced directional asymmetry: for the same discrepancy magnitude, the ELT > PLT condition exhibits a stronger mediated increase than ELT < PLT (relative indirect effect: b = 0.027, 95% CI [0.010, 0.043]). Taken together, these results support Hypothesis 4.

**Table 4:** Mediation effect analysis results.

Effect		Indirect	LLCI95%	ULCI95%
Congruence	Level	−0.033	−0.062	−0.004
Asymmetry	Absolute	0.017	0.001	0.034
	Relative	0.027	0.010	0.043

### 4.5 Research conclusions

The empirical findings of this study demonstrate that subordinates’ UI is significantly shaped by the interplay between ELT and PLT. First, when ELT and PLT are relatively aligned—indicating a high level of trust consistency—subordinates are less likely to engage in ingratiation. This suggests that alignment between trust expectations and perceptions helps reduce subordinates’ motivation to adopt impression management strategies.

Second, after controlling for trust consistency, the overall level of trust—reflected in the average of ELT and PLT—is positively associated with UI. Specifically, subordinates who strongly expect to be trusted, even if they only moderately perceive such trust, are more inclined to engage in ingratiatory behavior. This highlights the motivational power of trust expectations, which may drive subordinates to adopt strategic actions to gain or maintain desired trust.

Furthermore, the results reveal that, under equal levels of average trust and trust inconsistency, UI is more pronounced when ELT exceeds PLT, compared to the reverse pattern. This indicates that unmet trust expectations exert a stronger behavioral influence—subordinates who desire to be trusted but do not sufficiently perceive such trust are more likely to compensate through ingratiation.

In addition, mediation analyses indicate that trust consistency influences UI not only directly, but also indirectly through ARI. Higher trust consistency reduces ambivalence, thereby lowering the likelihood of UI; conversely, larger discrepancies between ELT and PLT increase psychological tension and promote ingratiatory tendencies. Notably, subordinates experiencing both high ELT and high PLT report the lowest levels of relational ambivalence and the weakest inclination toward ingratiation.

In sum, this study underscores the critical role of trust consistency in shaping subordinates’ strategic behaviors and reveals how different configurations of trust jointly influence the psychological and behavioral mechanisms underlying upward workplace interactions.

## 5 Discussion

To begin with, our findings demonstrate that congruence between ELT and PLT significantly reduces subordinates’ UI. This result aligns with the central premise of psychological contract theory (Rousseau and Tijoriwala, 1998), which suggests that consistency between individual expectations and organizational perceptions reduces the likelihood of compensatory behaviors.

After controlling for ELT–PLT discrepancies, we also found that higher overall trust—particularly elevated ELT—was positively associated with UI. This pattern reflects a tension between psychological safety and performance/relational pressure: high trust provides security and resources while simultaneously increasing role expectations and reciprocal obligations. To navigate this tension, employees engage in strategic behaviors, such as preventive ingratiation—proactively demonstrating cooperation, loyalty, and respect before formal evaluation—and actions aligned with the role-enhancement pathway, using being trusted as a role expectation and opportunity window to increase upward supportive behaviors, expressions of gratitude, and praise, thereby reinforcing role boundaries and strengthening upward relationships.

In contrast, when mean trust levels and absolute discrepancies are held constant, ingratiation is significantly more pronounced in scenarios where expected trust exceeds perceived trust. This asymmetric effect highlights the motivational power of unmet high expectations, underscoring the directional significance of misaligned cognitive evaluations (Humberg et al., 2022).

Turning to the underlying mechanisms, we find that trust congruence weakens subordinates’ ambivalent relational identification, which in turn reduces ingratiatory behavior. This finding is consistent with the dual-path model of social identity (Hogg and Terry, 2000), which posits that relational clarity and coherence stabilize the self-concept and reduce identity-related strain.

More importantly, our results reveal that high-trust congruence (i.e., high expected and perceived trust) exerts a stronger buffering effect on ambivalent identification and ingratiation than low-trust congruence (i.e., low expected and perceived trust). This nuanced insight advances the “trust-as-heuristic” perspective (Kramer, 1999), emphasizing that the quality of trust, rather than its mere presence, plays a pivotal role in shaping relational behavior.

Lastly, our study shows that trust incongruence intensifies ambivalent relational identification, which subsequently heightens upward ingratiation. This supports the mediating role of ambivalent identification within relational schema theory and extends its relevance to hierarchical trust dynamics in organizational settings (Ashforth et al., 2014).

Taken together, our research bridges the literature on trust with theories of social influence, proposing a dual-lens framework—focused on both trust congruence and trust level—to explain how trust calibration governs subordinates’ upward behavioral strategies.

### 5.1 Theoretical contributions

This framework yields four interrelated theoretical contributions.

To begin with, this study advances a dynamic interaction model of leadership trust. Prior research has predominantly adopted a unidimensional lens, either assessing the overall level of trust within leader-member dyads or focusing solely on an individual’s perception of being trusted. We depart from this paradigm by systematically integrating both ELT and PLT into an interactional framework and conceptualizing trust congruence—the degree to which subordinates’ expectations of being trusted align with their perceptions of actual leader trust. This shift reframes the central research question from “Is the leader trustworthy?” to “Does the employee feel their trust expectations are being met?” Theoretically, this model enhances the explanatory power of Leader-Member Exchange (LMX) theory by introducing trust alignment as a relational diagnostic tool that accounts for dynamic reciprocity in trust development (Brower et al., 2009). It also provides a new theoretical anchor for examining how subordinates cognitively appraise and behaviorally respond to shifting trust signals over time.

Building on this, our findings highlight the dual nature of trust in shaping employee behavior. Specifically, we find that higher aggregated trust levels (i.e., the mean of ELT and PLT) are positively associated with upward ingratiation, challenging the prevailing view that trust inherently inhibits strategic or self-serving actions. While trust fosters psychological safety, relational warmth, and access to resources, it can also generate pressure by implying expectations of reciprocity, loyalty, or consistent performance. In response, employees may engage in impression management—such as ingratiation—to preserve their relational standing. This insight extends Cognitive Evaluation Theory (Ryan et al., 1983) by framing trust not only as an emotional appraisal but also as a goal-oriented evaluation with both empowering and pressuring effects. These motivational tensions become particularly salient in contexts of moderate or incomplete trust, where employees are more likely to proactively shape perceptions to align with perceived expectations.

In addition, this study sheds light on how subordinates actively respond to trust incongruence through cognitive dissonance mechanisms, underscoring their strategic role in managing vertical relationships. When ELT exceeds PLT—reflecting a gap between expected and perceived trust—subordinates experience psychological discomfort or disequilibrium. To restore relational coherence and reaffirm their value, they are more likely to engage in upward ingratiation as a compensatory response. This challenges the traditional view of subordinates as passive recipients in trust exchanges and instead positions them as agentic actors who reshape social evaluations to alleviate cognitive strain. Drawing on Cognitive Dissonance Theory (Hinojosa et al., 2017), we conceptualize this response as a recursive process in which unmet trust expectations trigger psychological tension, prompting behavioral adjustment. This perspective extends dissonance theory into the domain of organizational trust and highlights the performative nature of employee behavior within hierarchical structures.

Finally, we propose a mediated psychological pathway through ARI to explain how trust congruence influences ingratiation via subordinates’ relational self-concept. When ELT and PLT are closely aligned, subordinates develop a stable and coherent relational identity with their leaders, reducing uncertainty and lowering the likelihood of impression management. In contrast, trust misalignment—particularly trust deficits—elicits emotional dissonance and relational ambiguity, weakening identification and increasing the need for compensatory behaviors. In such contexts, ingratiation functions as a coping mechanism to restore relational clarity and alleviate identity tension. This process entails cognitive reconciliation of trust incongruence, emotional regulation of relational strain, and the strategic enactment of impression management. By conceptualizing trust mismatch as a dynamic sequence of internal processing and external behavior, this study advances Relational Identification Theory (Sluss and Ashforth, 2007) and offers a more nuanced account of how subordinates manage identity tensions within hierarchical relationships, where trust carries both emotional and instrumental significance.

### 5.2 Managerial implications

This study offers several practical insights for management:

First, enhancing trust consistency should be a primary managerial goal. The consistency between subordinates’ expected trust from leaders and their perceived trust proves more effective in curbing strategic behaviors than trust level alone. Trust consistency reduces uncertainty and facilitates authentic, stable employee responses. Managers should therefore ensure that trust expressions are both perceptible and sustained. This involves articulating trust through explicit communication, empowerment, and emotional support, while maintaining behavioral congruence and transparency to avoid contradictions between verbal commitments and actual practices. Such consistency reinforces subordinates’ sense of being trusted, minimizes defensive behaviors, and fosters a trustworthy and cooperative organizational climate.

Second, trust may exert implicit pressure, requiring cautious application by managers. Contrary to conventional views that emphasize the benefits of high trust, our findings indicate that trust may also generate psychological burdens. When employees perceive high expectations of trust but fail to clearly experience such trust, they may feel obligated to justify that trust, resulting in increased performance pressure. This tension may prompt upward ingratiation or overcommitment to gain recognition. Hence, managers should adopt a more calibrated approach, attending to subordinates’ subjective trust perceptions and psychological responses. Establishing feedback channels and psychological safety mechanisms enables employees to express concerns, accept trust, and respond appropriately, promoting healthier leader–employee trust dynamics.

Third, managers should recognize and mitigate trust discrepancies. A notable gap where ELT exceeds PLT often leads to cognitive dissonance and psychological discomfort, triggering compensatory strategic behaviors. Such “trust gaps” can weaken emotional bonds with the organization and increase relational anxiety, encouraging impression management behaviors to restore perceived trust. Managers must therefore identify employees experiencing trust discrepancies and engage in timely one-on-one communication, emotional coaching, and affirming feedback. Concrete actions—such as delegated responsibilities and specific praise—can enhance the visibility of trust, alleviating uncertainty and fostering authentic, trust-based relationships.

Finally, fostering subordinates’ relational identification can fundamentally reduce strategic behaviors. Relational identification refers to the emotional and identity-based alignment employees develop with their leaders. This study finds that trust consistency strengthens such identification, thereby indirectly reducing employees’ tendency to engage in strategic behaviors. When employees perceive leadership trust as both genuine and sustained, they are more likely to internalize the leader’s values and voluntarily maintain harmonious leader–member relationships. To cultivate this, managers should go beyond structural trust-building and attend to the emotional dimension—clarifying role boundaries and interactional expectations. Practices such as encouraging two-way communication, expressing emotional support, and promoting a shared relational culture help employees define their identity and behavioral norms, mitigating motivation distortion due to role ambiguity and enabling intrinsically driven, authentic behavior.

### 5.3 Limitations and prospects

Although this study has made meaningful contributions to understanding the relationship between trust congruence and employee behavior, several limitations remain, which also offer potential directions for future research.

First, this study adopted a cross-sectional design, which limits the ability to draw causal inferences. Although we developed a theoretically rigorous model grounded in role theory to support the proposed pathway through which trust congruence affects employee behavior, cross-sectional data are inherently limited in capturing the dynamic nature of variables over time. Future research may employ longitudinal designs to examine how trust congruence evolves and accumulates its effects on employee behavior across different time points. Additionally, experimental methods, such as scenario-based simulations or controlled experiments, could be valuable in enhancing causal inference and further validating the underlying mechanisms proposed in this study.

Second, although this study utilized a multi-wave, multi-source data collection approach—where supervisors rated subordinates’ ingratiation behaviors while other variables were self-reported by employees—certain limitations remain. Specifically, self-reported data may still be subject to social desirability bias and self-presentation effects, especially for subjective constructs such as perceived trust. Moreover, discrepancies in perceptions across data sources may introduce cognitive biases that affect the observed relationships. To further enhance the validity and reliability of findings, future studies could incorporate additional sources of data, such as coworker ratings, behavioral observations, or objective performance indicators, to provide a more comprehensive and accurate assessment of trust congruence and its behavioral consequences.

Third, although this study focuses on the dual effects of ELT and PLT, it does not fully account for the potential moderating role of contextual factors. Organizational culture, team climate, leadership style, and job autonomy may influence the relationship between trust configurations and employee behaviors. For instance, a highly inclusive organizational culture may buffer the negative effects of trust inconsistency, whereas a controlling leadership style may exacerbate employee anxiety and role conflict. In addition, leader prototypicality and employees’ perceptions of decision fairness may serve as critical boundary conditions: highly prototypical leaders may encourage upward ingratiation, while high fairness perceptions may strengthen relational identification and reduce strategic ingratiation. Future research could incorporate these variables as controls or cross-level moderators to examine their interaction with ELT–PLT trust configurations, thereby more precisely identifying underlying mechanisms and enhancing external validity.

Finally, this study examined ingratiation behavior as the focal outcome; however, trust congruence, as a form of role perception mechanism, is likely to have broader implications. Future research may expand the outcome scope by investigating other trust-related employee behaviors, such as voice behavior, organizational citizenship behavior (OCB), psychological detachment, or turnover intentions. These outcomes not only have important implications for individual development but are also closely tied to organizational innovation and sustainability. By incorporating a broader array of outcome variables, future studies can further enrich the theoretical framework of trust congruence and enhance its practical relevance.

## 6 General conclusion

Managers should move beyond the traditional notion of “the higher the trust, the better” and adopt a more dynamic understanding and precise regulation of “trust configurations” and “trust processes.” In today’s complex and rapidly changing organizational environments, trust is no longer a static, one-dimensional resource; rather, it is a dynamic, co-constructed, and bidirectionally regulated process. Only by understanding employees’ subjective trust perceptions and their psychological coping mechanisms can managers effectively calibrate the form, frequency, and mode of trust expression. This nuanced approach enables the stabilization of organizational relationships and the positive guidance of employee behavior. Accordingly, future managerial practices should place greater emphasis on the quality of employees’ trust experience and aim to build resilient and adaptive leadership trust mechanisms—thereby fostering the development of efficient, healthy, and sustainable organizations.

## Data Availability

The raw data supporting the conclusions of this article will be made available by the authors, without undue reservation.
